# *Wolbachia* prevalence in the vector species *Culex pipiens* and *Culex torrentium* in a Sindbis virus-endemic region of Sweden

**DOI:** 10.1186/s13071-021-04937-6

**Published:** 2021-08-26

**Authors:** Alexander Bergman, Jenny C. Hesson

**Affiliations:** Department of Medical Biochemistry and Microbiology, Zoonosis Science Center, Uppsala University, Uppsala, Sweden

**Keywords:** Vector, Field, Mosquito, Endosymbiont, Alphavirus, Horizontal transmission, Scandinavia

## Abstract

**Background:**

*Wolbachia pipientis* are endosymbiotic bacteria present in a large proportion of terrestrial arthropods. The species is known to sometimes affect the ability of its host to transmit vector-borne pathogens. Central Sweden is endemic for Sindbis virus (SINV), where it is mainly transmitted by the vector species *Culex pipiens* and *Culex torrentium*, with the latter established as the main vector. In this study we investigated the *Wolbachia* prevalence in these two vector species in a region highly endemic for SINV.

**Methods:**

*Culex* mosquitoes were collected using CDC light traps baited with carbon dioxide over 9 years at 50 collection sites across the River Dalälven floodplains in central Sweden. Mosquito genus was determined morphologically, while a molecular method was used for reliable species determination. The presence of *Wolbachia* was determined through PCR using general primers targeting the *wsp* gene and sequencing of selected samples.

**Results:**

In total, 676 *Cx. pipiens* and 293 *Cx. torrentium* were tested for *Wolbachia*. The prevalence of *Wolbachia* in *Cx. pipiens* was 97% (95% CI 94.8–97.6%), while only 0.7% (95% CI 0.19–2.45%) in *Cx. torrentium*. The two *Cx. torrentium* mosquitoes that were infected with *Wolbachia* carried different types of the bacteria.

**Conclusions:**

The main vector of SINV in the investigated endemic region, *Cx. torrentium*, was seldom infected with *Wolbachia*, while it was highly prevalent in the secondary vector, *Cx. pipiens*. The presence of *Wolbachia* could potentially have an impact on the vector competence of these two species. Furthermore, the detection of *Wolbachia* in *Cx. torrentium* could indicate horizontal transmission of the endosymbiont between arthropods of different species.

**Graphical abstract:**

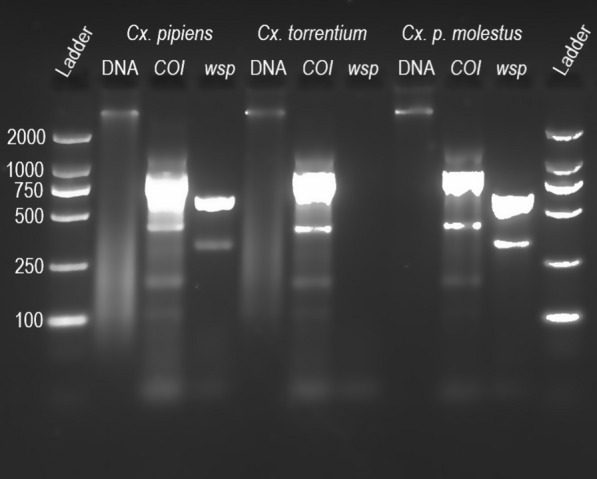

## Background

The transmission of arboviruses is influenced by a number of factors, including both abiotic (e.g., temperature) and biotic elements (e.g., vector immune status) [1–4]. One important biotic factor is the intracellular symbiont *Wolbachia pipientis* (Class: Alphaproteobacteria, Order: Rickettsiales), present in some nematode species and an estimated 40% of all terrestrial arthropods [5]. *Wolbachia* is a genetically diverse species, composed of 18 phylogenetically distinct supergroups described to date (A–R) [6]. Deeply involved in the reproduction of its host [7], *Wolbachia* is known for inducing cytoplasmic incompatibility and giving rise to crossing types, most studied in *Culex pipiens* and its *Wolbachia* strain *w*Pip, which belongs to supergroup B [8–10]. Additionally, it is well established that *Wolbachia* infection in mosquitoes can influence their ability to become infected and transmit several arboviruses [11–14].

In a global context, *Culex* mosquitoes are important vectors for, e.g., West Nile virus (WNV) and Japanese encephalitis virus (JEV) [15–18]. In Central and Northern Europe, the morphologically identical vector species *Cx. pipiens* and *Cx. torrentium* are enzootic vectors of both WNV and Sindbis virus (SINV), transmitting these viruses among birds [19–22]. SINV is an arthritogenic alphavirus present throughout the Old World [23], although outbreaks of human disease are only reported from South Africa [24, 25] and Fennoscandia [26–29]. In Sweden, SINV is considered endemic to the central and northern parts of the country [28–30].

*Culex torrentium* is regarded as the most important enzootic vector in Sweden due to its high abundance in endemic areas, high infection rate, and superior vector competence to *Cx. pipiens* [31–34]. One difference between *Cx. torrentium* and *Cx. pipiens* is the prevalence of *Wolbachia*-infected individuals. Previous studies in Germany, Belgium, Russia, Belarus, Kazakhstan, and Kyrgyz Republic have found *Wolbachia* to be very common in *Cx. pipiens* but absent in *Cx. torrentium* [35–38]. It is therefore possible that these differences in *Wolbachia* infection status could account for part of the difference in vector competence seen between *Cx. pipiens* and *Cx. torrentium*. Previous studies have however only been performed in regions without intense SINV transmission. Therefore, this study aims at investigating the *Wolbachia* prevalence in *Cx. pipiens* and *Cx. torrentium* collected in a highly SINV-endemic region in central Sweden.

## Methods

### Mosquitoes

**Fig. 1:**
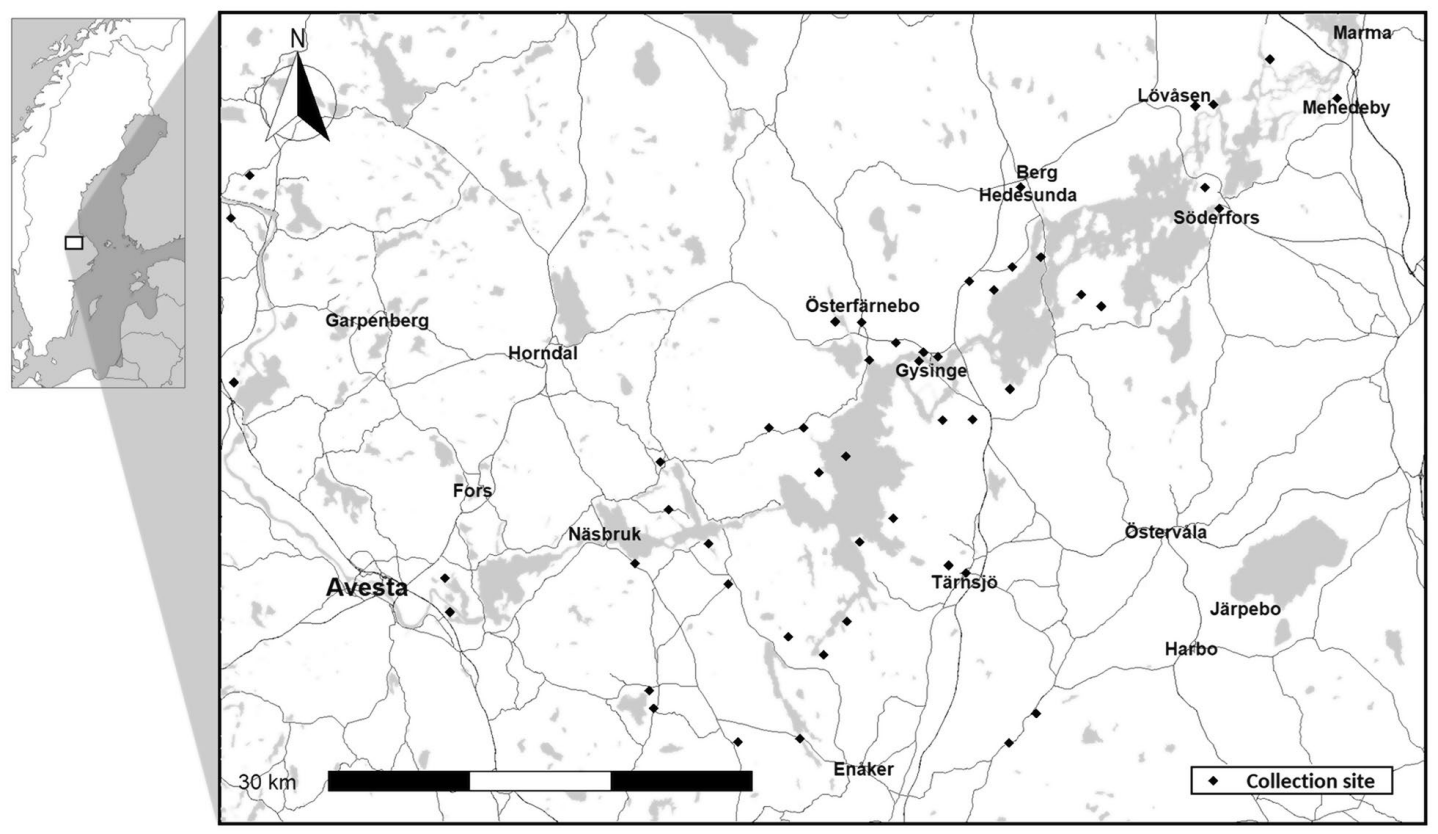
Map showing the collection sites of *Wolbachia*-screened *Culex* mosquitoes. Collection sites are marked as black diamonds. Map data retrieved from ^©^OpenStreetMap contributors under the Open Database License

Mosquitoes were collected at 50 different locations across the River Dalälven floodplains (Fig. 1) as part of a routine mosquito monitoring programme [39]. SINV is considered endemic to this region and some of the highest infection rates in mosquitoes have been detected here [22, 33]. Collections were performed every second week between May and September during the years 2010–2018 using CDC light traps baited with carbon dioxide. Mosquitoes were identified based on morphological characteristics [40], and *Cx*. *pipiens*/*torrentium* were sorted out and used for molecular identification to species. Briefly, individual mosquitoes were homogenized in 500 µl of phosphate-buffered saline (PBS) supplemented with 20% heat-inactivated fetal bovine serum, 100 U/ml penicillin, 100 μg/ml streptomycin, and 2.5 μg/ml amphotericin B (Thermo Fischer Scientific; Waltham, MA, USA) using two steel beads in the Qiagen TissueLyser II™ (Qiagen; Hilden, Germany). Five microliters (5 µl) of the homogenate was pretreated by incubating at 98 °C for 2 min in 20 µl of dilution buffer with 0.5 µl of DNA release additive, part of the Phire Tissue Direct PCR Master Mix kit (Thermo Scientific; Vilnius, Lithuania). The pretreated homogenate was stored at −20 °C before being used as a template in polymerase chain reaction (PCR). Conventional PCR of part of the cytochrome oxidase subunit I (*COI*) was performed in 20 µl reactions with 1 µl template using the forward primer C1-J-2183 (5′-CAACATTTATTTTGATTTTTTGG-3′) and the reverse primer TL2-N-3014 (5′-TCCAATGCACTAATCTGCCATATTA-3′) at a concentration of 0.5 µM each under the following thermocycler conditions: initial denaturation at 98 °C for 5 min, followed by 40 cycles of denaturation at 98 °C for 5 s, annealing at 54.5 °C for 5 s and extension at 72 °C for 20 s, and a final extension step at 72 °C for 1 min. A PCR-restriction fragment length polymorphism (PCR–RFLP) assay [41] was performed on the PCR product, using the restriction enzymes FspBi and SspI (Thermo Fischer Scientific; Vilnius, Lithuania).

*Culex pipiens molestus* mosquitoes, originating from a field population sampled in Gothenburg, Sweden [42] and reared in our in-house mosquito rearing facility, were used as positive controls in PCR as they are naturally infected with a *w*Pip strain of *Wolbachia* (data not shown). These were also used for PCR optimization.

### *Wolbachia* detection

**Fig. 2:**
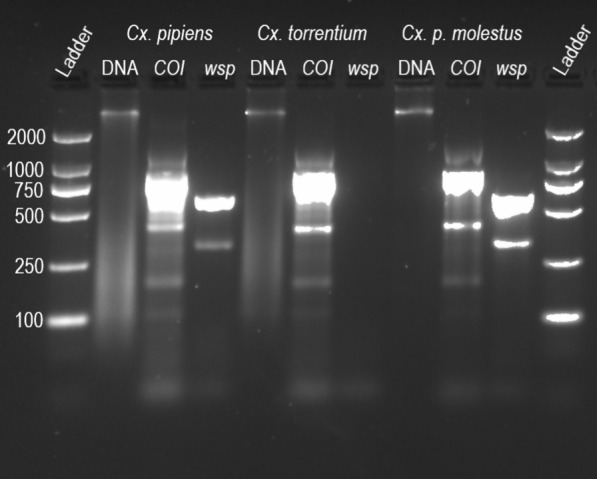
Representative agarose gel of extracted DNA and amplified PCR products. The extracted DNA (lanes 2, 5, and 7), amplified cytochrome oxidase subunit I (*COI*) gene (lanes 3, 6, and 9), and amplified *wsp* gene (lanes 4, 7, and 10) are shown for an individual of each of the species *Cx. pipiens*, *Cx. torrentium*, and *Cx. pipiens molestus*, respectively

*Wolbachia* detection was performed through PCR on 5 µl of the mosquito homogenate, using the same Tissue Direct kit procedures as described above. *Wolbachia* primers 81F (5′-TGGTCCAATAAGTGATGAAGAAAC-3′) and 691R (5′-AAAAATTAAACGCTACTCCA-3′), designed for general detection of *Wolbachia* within supergroups A and B [43], were used at a final concentration of 0.5 µM each. The thermocycler conditions for *Wolbachia* detection were as described above but with the annealing temperature set to 58 °C. A subset of samples was also tested with a confirmatory PCR to determine whether the detected *wsp* gene *Wolbachia* belonged to that of the *w*Pip strain using *w*Pip-specific primers wPF (5′-CGACGTTAGTGGTGCAACATTTA-3′) and wPR (5′-AATAACGAGCACCAGCAAAGAGT-3′) [44] with the same PCR conditions as described previously but with the annealing temperature set to 56 °C. For primer optimization, DNA integrity was controlled by extraction of total DNA to make sure that a negative PCR result was not due to DNA degradation in the sample. DNA was extracted from 44 samples with the E.Z.N.A.^®^ Tissue DNA Kit (Omega Bio-Tek, Inc., Norcross, GA, USA), and visual inspection of DNA integrity was done by gel electrophoresis. Extracted DNA and all PCR products were visualized on 1.8% agarose gel stained with GelRed^®^ Nucleic Acid Gel Stain (Biotium, Fremont, CA, USA) (Fig. 2). A subset of PCR products was purified with ExoSAP-IT^®^ (Thermo Fischer Scientific; Vilnius, Lithuania) and sequenced through Sanger sequencing (Macrogen; Amsterdam, The Netherlands) to validate the method and verify the results.

### Data analysis

All records were kept and analysed in Microsoft Excel 2016 (Microsoft; Redmond, CA, USA). Confidence intervals for *Wolbachia* prevalence were calculated assuming binomial distribution using the Wilson score interval through RStudio (RStudio team, Boston, USA). *P*-values to determine statistical significance for differences in *Wolbachia* prevalence between years were calculated using Fisher’s exact test with Bonferroni correction. Sequences of PCR fragments were analysed in the BioEdit sequence alignment editor version 7.2.5 [45].

## Results

In total, 969 *Culex* mosquitoes (676 *Cx. pipiens* and 293 *Cx. torrentium*) were identified to species and tested for *Wolbachia* (Fig. 2). *Wolbachia* was present in 96.5% of the *Cx. pipiens* population (95% CI 94.8–97.6%) but could only be detected in two out of 293 *Cx. torrentium* individuals (0.68% prevalence, 95% CI 0.19–2.45%) (Table 1). Three of the *Cx. pipiens* that carried *Wolbachia* from each year were tested with primers specific to the *w*Pip variant of *wsp*, of which all 27 were found to carry a *wsp* belonging to the *w*Pip strain. In 2012, the prevalence of *Wolbachia* in *Cx. pipiens* was significantly lower than normal (Fisher’s exact test: *P * =  0.00455, OR: 0.389 CI [0.198–0.778], Bonferroni-corrected *P * =  0.041).

**Table 1:** Results of the screening of *Cx. pipiens* and *Cx. torrentium* for *Wolbachia*

Year	Species						Total tested
	*Culex pipiens*			*Culex torrentium*			
	Tested	Positive	% positive	Tested	Positive	% positive	
2010	93	92	98.9	49	0	0	142
2011	21	21	100	30	0	0	51
2012	208	190	91.3	71	0	0	279
2013	62	60	96.8	52	0	0	114
2014	6	5	83.3	2	0	0	8
2015	76	75	98.7	39	2	5.2	115
2016	30	30	100	30	0	0	60
2017	53	53	100	10	0	0	63
2018	127	126	99.2	10	0	0	137
Total	676	652	96.5	293	2	0.7	969

The mosquitoes were collected in central Sweden between 2010 and 2018. The prevalence of *Wolbachia* in *Cx. pipiens* differed significantly in year 2012 from the 9-year average (Fisher’s exact test: *P * =  0.00455, OR: 0.389 CI [0.198–0.778], Bonferroni-corrected *P * =  0.041). The differences for all other years are non-significant

**Fig. 3:**
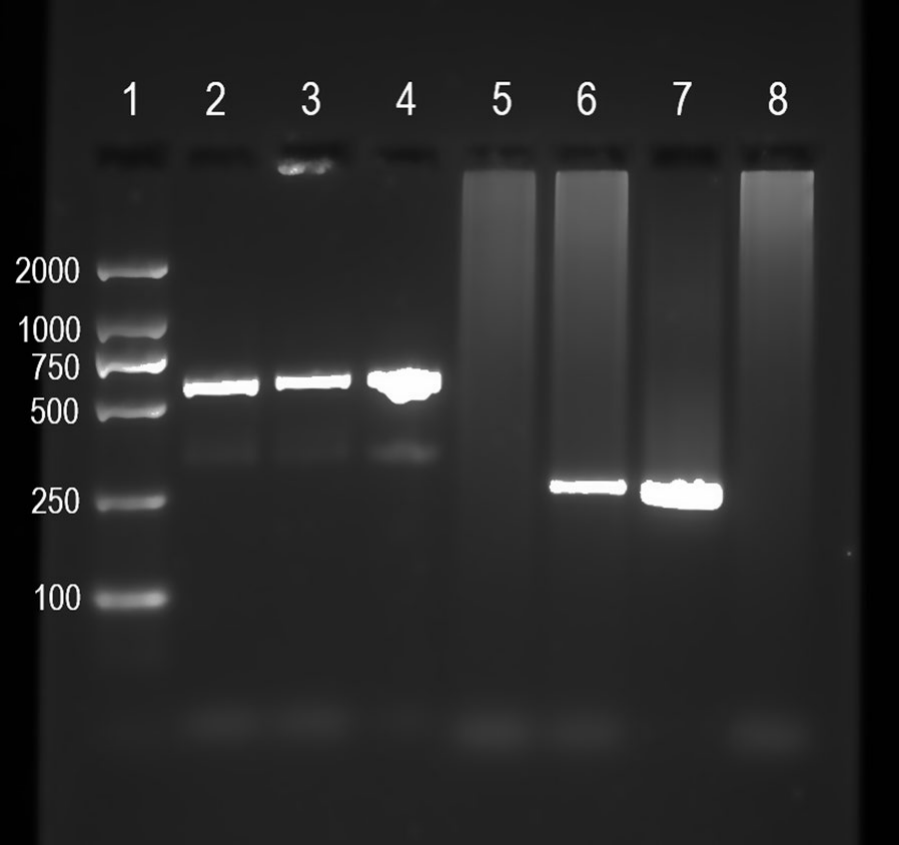
Gel showing amplification with general *wsp* primers (*Cx. torrentium* in lanes 2–3, *Cx. pipiens molestus* in lane 4), and with specific *w*Pip primers (*Cx. torrentium* in lanes 5–6, *Cx. pipiens molestus* in lane 7). Lane 1 shows a size ladder and lane 8 shows a combined non-template control for general and specific *wsp* amplification

Two *Cx. torrentium* were found to carry *Wolbachia*. Sequencing of the amplicons showed that the two partial *wsp* sequences were only 90% identical to each other. The *wsp* sequence from one of the *Cx. torrentium* individuals was very similar (>  99.8% identity) to the *wsp* of *Wolbachia* from *Cx. pipiens* (GenBank: KT964224.1), but also to isolates from the winter moth (*Operophtera brumata*: GenBank: KY587652.1), cabbage moth (*Mamestra brassicae*; GenBank: AB094375.1), and *Toya propinqua* (GenBank: KM386826.1). The other *Cx. torrentium* carried a *Wolbachia* whose *wsp* gene was highly similar (>  99.6% identity) to that of *Wolbachia* detected in several other insects, namely the spotted fritillary (*Melitaea didyma*; GenBank: MN322891.1), silverleaf whitefly (*Bemisia tabaci*; GenBank: AJ291379.1), azalea lace bug (*Stephanitis pyrioides*, GenBank: AB109622.1), *Macrolophus pygmaeus* (GenBank: FJ374283.1), and *Amaurosoma flavipes* (GenBank: JN601166.1), all of which carry *Wolbachia* from supergroup B. The sequencing results were confirmed by PCR using the *w*Pip-specific *wsp* primers. This PCR amplified a correct fragment from only one of the two *Wolbachia*-positive *Cx. torrentium* (Fig. 3).

## Discussion

We found that *Wolbachia* was highly prevalent in *Cx. pipiens* collected around the River Dalälven floodplains, while it was nearly absent from *Cx. torrentium*. This is in line with previous European studies investigating large samples of *Cx. pipiens*, with reported *Wolbachia* prevalence of 91% in western Russia [35], 95% in central Russia, 81% in Belarus [36], and 93% in Germany [37]. Raharimalala et al. [38] detected *Wolbachia* in nine out of nine tested adult *Cx. pipiens* and 26 out of 48 larvae collected in Belgium, which also supports the generally high prevalence of *Wolbachia* in this species. Interestingly, the different populations studied by Khrabrova et al. [36] had varying levels of *Wolbachia* prevalence, some with as few as 34.5% of individuals carrying the endosymbiont. *Wolbachia* is reported to approach fixation in most *Cx. pipiens* populations worldwide [46, 47], but this does not seem to hold true for all European populations.

Only Ricci et al. [48] have, to our knowledge, previously found *Wolbachia* in *Cx. torrentium,* after testing only two individuals collected in Italy. Raharimalala et al. [38], Leggewie et al. [37], Vinogradova et al. [35], and Khrabrova et al. [36] detected no *Wolbachia* in *Cx. torrentium* despite having tested 42 Belgian, 188 German, 321 Russian, and 853 Eastern European individuals, respectively. Our study, as well as the study by Ricci et al. [47], tested adult mosquitoes, while the four that failed to detect *Wolbachia* in *Cx. torrentium* tested field-collected larvae and pupae. *Wolbachia* is usually inherited and should thus be present in all life stages of the mosquito; however, life stage is still potentially an important consideration when screening for *Wolbachia*, both to avoid analysing siblings and to detect potential horizontal transmission.

Due to the low prevalence of *Wolbachia* in *Cx. torrentium*, we hypothesize that the two positive individuals or their recent ancestors acquired the infection horizontally. Transmission could potentially have occurred by feeding on the same plants as other arthropods [49, 50] or through arthropod parasites, such as through mites sometimes feeding on mosquitoes [51, 52]. Despite *wsp* being a poor marker of *Wolbachia* strain due to its tendency to recombine [53], the lineage of the *Cx. torrentium* whose *wsp* gene matched that of the *w*Pip strain could have acquired its infection from a *Cx. pipiens* through their shared habitat and ecological niche. Alternative sources are also possible, since a highly similar *wsp* sequence has also been found in other Palearctic insects. Further studies on the mechanisms for horizontal *Wolbachia* transmission involving mosquitoes are needed to fully explain the occasional spread of *Wolbachia* to *Cx. torrentium*.

The restriction of SINV outbreaks to Northern Europe has been suggested to be connected to the relatively higher abundance of the competent vector species *Cx. torrentium* in SINV-endemic regions [31, 32]. Under laboratory conditions, *Cx. torrentium* is significantly more susceptible to SINV infection than *Cx. pipiens* [34, 54]. The presence of *Wolbachia* in *Cx. pipiens* may contribute to its lower susceptibility to SINV. Such reduction in vector competence is often seen when transferring a novel *Wolbachia* strain into a mosquito species that is naturally *Wolbachia*-free or naturally carries a different strain [11, 55–58], but the impact of a naturally occurring *Wolbachia* infection (i.e., native infection) is not as clear, with reports of both reduced vector competence [13, 14, 59] and no observed effect [60–63]. No vector competence studies have been done on the role of *Wolbachia* in alphavirus transmission in *Culex* mosquitoes. With relatively few data to extrapolate from, empirical investigation is needed to evaluate the impact of *Wolbachia* on the SINV transmission cycle.

## Conclusions

Our study, performed in a SINV-endemic region of Sweden, confirmed previously reported general patterns of *Wolbachia* infection in *Culex* mosquitoes, with most *Cx. pipiens* and very few *Cx. torrentium* carrying the endosymbiont, which potentially has implications for their differences in vector competence. Our findings, paired with the specific conditions under which SINV is transmitted in Sweden, prompt more research into *Wolbachia*’s role in the SINV transmission cycle as well as the horizontal routes of *Wolbachia* transmission among mosquitoes.

## Data Availability

For amplicon sequences of the *Wolbachia* detected in *Cx. torrentium* and C*x. pipiens*, the data sets generated and/or analysed during the current study are available in the GenBank repository, accession numbers MW622245-MW622247. All other data sets used and/or analysed during the current study are available from the corresponding author on reasonable request.

## Abbreviations

CDC: Centers for Disease Control and Prevention
CHIKV: Chikungunya virus
*COI*: Cytochrome oxidase subunit I
*Cx.*: *Culex*
JEV: Japanese encephalitis virus
PBS: Phosphate buffered saline
PCR–RFLP: Polymerase chain reaction–restriction fragment length polymorphism
SINV: Sindbis virus
WNV: West Nile virus
